# Juvenile hormone reveals mosaic developmental programs in the metamorphosing optic lobe of *Drosophila melanogaster*

**DOI:** 10.1242/bio.034025

**Published:** 2018-04-15

**Authors:** Lynn M. Riddiford, James W. Truman, Aljoscha Nern

**Affiliations:** Janelia Research Campus, Howard Hughes Medical Institute, 19700 Helix Drive, Ashburn, VA 20147, USA

**Keywords:** Juvenile hormone, Ecdysone, Optic lobe, *Drosophila melanogaster*

## Abstract

The development of the adult optic lobe (OL) of *Drosophila melanogaster* is directed by a wave of ingrowth of the photoreceptors over a 2-day period at the outset of metamorphosis, which is accompanied by the appearance of the pupal-specific transcription factor Broad-Z3 (Br-Z3) and expression of early drivers in OL neurons. During this time, there are pulses of ecdysteroids that time the metamorphic events. At the outset, the transient appearance of juvenile hormone (JH) prevents precocious development of the OL caused by the ecdysteroid peak that initiates pupariation, but the artificial maintenance of JH after this time misdirects subsequent development. Axon ingrowth, Br-Z3 appearance and the expression of early drivers were unaffected, but aspects of later development such as the dendritic expansion of the lamina monopolar neurons and the expression of late drivers were suppressed. This effect of the exogenous JH mimic (JHM) pyriproxifen is lost by 24 h after pupariation. Part of this effect of JHM is due to its suppression of the appearance of ecdysone receptor EcR-B1 that occurs after pupation and during early adult development.

## Abstract (precis)

**Summary:** Developmental gradients and steroid surges interact during optic lobe development. Early, ectopic juvenile hormone treatment alters steroid receptor levels, suppresses late events but not early events linked to developmental gradients.

## INTRODUCTION

In insects, ecdysone (a steroid hormone) causes molting and metamorphosis and juvenile hormone (JH) (a sesquiterpenoid hormone) prevents metamorphosis and is critical for the normal growth and development of the immature stage (Nijhout, 1995; Truman et al., 2006). In most insects larval cells are reprogrammed for metamorphosis by ecdysone acting in the absence of JH (Riddiford, 1994). By contrast, in *Drosophila* larval cells die except in the nervous system and the Malpighian tubules, and the adult is formed from imaginal discs and imaginal precursor cells (Fristrom and Fristrom, 1993). Unlike many insects, *Drosophila* larvae are mosaic in terms of their sensitivity to JH. For epidermal structures, for example, the imaginal discs are insensitive to JH, whereas the abdominal histoblasts that make the adult cuticle are sensitive (Postlethwait, 1974; Zhou and Riddiford, 2002). In general, the adult nervous system is sensitive to JH given at pupariation or during the prepupal period (Restifo and Wilson, 1998; Riddiford et al., 2010), but, interestingly, this nervous system is interacting with sensory neurons that are growing in from imaginal discs that are insensitive to JH.

In *Drosophila melanogaster*, the lack of JH due to the genetic ablation of the corpora allata (CA), the gland that produces JH, causes slow growth in the final larval stage (Mirth et al., 2014) and death at the time of the pupal molt (Liu et al., 2009; Riddiford et al., 2010). These allatectomized larvae, however, showed precocious optic lobe development during the prepupal period including premature decrease of proliferation in the inner and outer proliferation zones, appearance of the ecdysone receptor (EcR)-B1, and separation of the photoreceptors R7 and R8 terminals in the medulla (Riddiford et al., 2010). By contrast, application of exogenous JH at pupariation suppressed the appearance of EcR-B1 and delayed the cessation of proliferation and the separation of the R7 and R8 terminals. *Drosophila* has two closely related JH receptors, Methoprene-tolerant (Met) and Germ cells-expressed (Gce), both of which must be absent to cause death at the pupal molt (Abdou et al., 2011). Interestingly, the *Met*-null mutant showed the same precocious development of the optic lobe as the allatectomized larvae, which resulted in mis-shaping of the lobula and disruption of the dendritic layering of this adult neuropil (Riddiford et al., 2010). By contrast, the *gce*-null mutant had no effect on optic lobe development (Riddiford, 2012). We now know that this difference is due to only Met being present in the optic lobe at pupariation (Baumann et al., 2017).

The adult optic lobe of *D.* *melanogaster* consists of four neuropils: lamina, medulla, lobula and lobula plate. Its development is directed by incoming photoreceptors from the eye (Meinertzhagen and Hanson, 1993; Ting and Lee, 2007). At the mid-third larval instar, the photoreceptors begin to differentiate in each ommatidium behind the morphogenetic furrow as it moves across the eye imaginal disc (Wolff and Ready, 1993). By the time of head eversion to the pupa, 10-12 h after pupariation (APF), all the ommatidia have begun differentiation and the photoreceptors have sent their axons into the optic lobe. Photoreceptors R1-R6 synapse with the developing lamina neurons, whereas R7 and R8 grow directly into the medulla (Meinertzhagen and Hanson, 1993). The lamina neurons subsequently grow into and arborize in the medulla (Fischbach and Dittrich, 1989; Ting et al., 2005; Nern et al., 2008).

This progressive patterning of the adult optic lobe that begins with the wave of differentiation of the photoreceptors across the eye seems to do so smoothly through the onset of metamorphosis when there are dramatic hormonal changes in both ecdysone and JH titers. Although EcR is present in the eye imaginal disc during the third larval instar, its absence had no effect on the initiation and the normal movement of the morphogenetic furrow (Brennan et al., 2001). Similarly, the absence of JH in the third instar had no effect on the movement of the furrow as indicated by the ingrowth of the photoreceptors into the lamina (Riddiford et al., 2010). Thus, furrow movement and the determinative events that follow occur according to a developmental clock that appears to ignore the dramatic changes in hormonal titers that provoke degeneration of larval tissues and the organization and differentiation of the adult form. Yet, as described above, the absence of JH at the time of the pupariation peak of ecdysteroid allowed at least some aspects of adult development of the optic lobe to occur precociously during the prepupal period (Riddiford et al., 2010). In contrast, a persistent JH mimic (JHM) applied at pupariation delayed these aspects of development. Thus, JH must be present at the time of pupariation to prevent the differentiative effects of high ecdysteroid during the prepupal period. However, its presence must be transient to allow normal differentiation in response to the adult ecdysteroid peak.

To determine how the developing adult optic lobe deals with these two conflicting developmental programs – one clocklike tied to the movement of the morphogenetic furrow and the other hormone-dependent on both ecdysteroid and JH titers, we have studied the responsiveness of different neuronal types to JH given during the prepupal and early pupal period. We find that these cell types differ dramatically in their sensitivity to JH treatment. For the sensitive ones, the developmental disruptions were found to be partially dependent on the JH-induced suppression of EcR-B1 but other unknown factors are involved as well.

## RESULTS

**Fig. 1.**
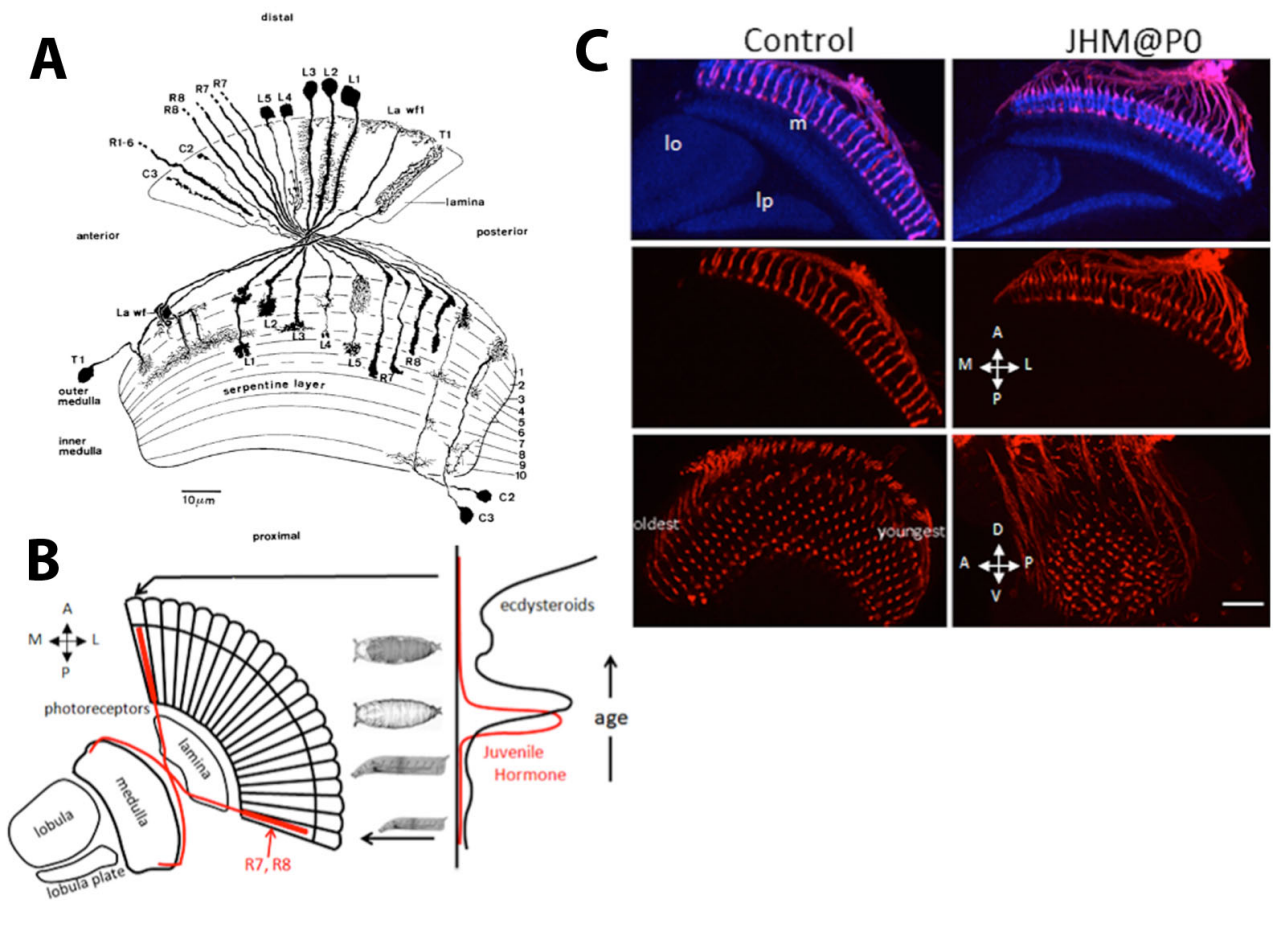
**Diagram of neurons in the outer optic lobe and the effect of JHM on photoreceptor ingrowth.** (A) Camera lucida drawings of the photoreceptor axons (R1-R8) and the neurons innervating the lamina and the medulla of the adult optic lobe of *D.* *melanogaster.* See text for the description of the various neurons. (B) Diagram illustrates the progressive ingrowth of R7 and R8 into the medulla of the optic lobe over time at the onset of metamorphosis when the ecdysteroid and JH titers are fluctuating. (C) Effect of JHM on the ingrowth of R7 and R8 photoreceptors into the medulla. The JHM pyriproxifen was applied at the time of pupariation (P0) and the optic lobes of uneclosed day 1 adults were imaged (*n*=16). The controls were untreated day 1 adults (*n*=4). The images are sections viewed along the dorsoventral (D,V) axis (top and middle) and along the medial-lateral (M,L) axis (bottom). Red, chaoptin; blue, N-cadherin. m, medulla; lo, lobula; lp, lobula plate. Scale bar: 50 µm. Fig. 1A is Fig. 3A in Fischbach and Dittrich (1989) in *Cell and Tissue Research,* reprinted by permission from Nature/Springer/Palgrave.

Fig. 1A shows the lamina and medulla of the adult optic lobe of *Drosophila* and the neurons that innervate these two neuropils (Fischbach and Dittrich, 1989). The neuropils are organized into discrete lamina cartridges and medulla columns that are organized around sets of photoreceptors. The photoreceptors R1-R6 terminate in the lamina cartridges, while R7 and R8 extend into the medulla columns. The medulla contains the greatest number of neuronal types in the optic lobe. It has a layered neuropil that is organized into vertical columns, each corresponding to an ommatidium (Fischbach and Dittrich, 1989). The anterior medulla columns are the oldest and are innervated by the R7 and R8 axons from the most posterior ommatidia and by lamina interneurons from the most posterior cartridges, while the most posterior medulla columns form from axons from the youngest ommatidia and cartridges.

The output cells of the lamina are the five lamina monopolar neurons (L1-L5) that have terminal arbors in different layers of the outer medulla (Fig. 1A). Three of the cells have two distinct arbors in different layers of the medulla: L1 has a distal arbor in layer 1 and a terminal arbor in layer 5, L4 arborizes in both layers 2 and 4, and L5 has a terminal arbor in layer 5 and a distal arbor in layer 1 extending into layer 2. We use the term ‘distal’ to refer to the outer medulla layers M1-M6 and ‘proximal’ to refer to the inner layers M7-10 as used by Fischbach and Dittrich (1989). The lamina wide-field 1 (Lawf1, called Lawf in Fischbach and Dittrich, 1989) neurons arborize at the surface of the medulla and also in layer 3; their axons also extend into the lamina where they arborize on the surface. The T1 neurons in the medulla cortex arborize in layer 2 of the medulla and then project to the lamina where they extend broadly through this neuropil. Both the C2 and C3 centrifugal neurons grow in from the cell body rind near the posterior inner medulla and arborize in at least three layers of the medulla with the most prominent being in layer 1, and finally terminate in the lamina. The transmedullary Tm1 neurons from the medulla cortex arborize in layers 2/3 and 9 of the medulla, then terminate in the outer layer of the lobula (not shown in Fig. 1A; see Fischbach and Dittrich, 1989). The T4 and T5 neurons arise just posterior to the lobula plate, grow through the lobula plate to arborize in the innermost layer of the medulla (T4) or the outer layer of the lobula (T5), and then project back to the lobula plate (not shown in Fig. 1A; see Fischbach and Dittrich, 1989).

### JH mimic effects on the ingrowth of photoreceptors R7 and R8

Photoreceptors R1-R6 extend into the optic lobe and form a densely packed array in the lamina cartridges (Fig. 1A). Photoreceptors R7 and R8 extend through the lamina and cross a chiasm into the medulla where they are spaced out in a crystalline-like array in the upper layers of the medulla. Because of the chiasm, the first-born R7 and R8 from the posterior margin of the eye terminate in the most anterior margin of the medulla with the later-born axons assuming progressively posterior positions in the medulla. As the diagram in Fig. 1B shows, the ingrowth of photoreceptor R8 axons from the developing ommatidia begins at the mid-third instar, continues into the early pupal period and is finished by about 24 h APF (Kulkarni et al., 2016). The R7 axon ingrowth from a given ommatidium follows about 6-8 h later. When the JH mimic (JHM) pyriproxifen was applied at the time of pupariation, the orderly progression of R7 and R8 ingrowth was not affected (Fig. 1C). However, their axon terminals showed some disarray rather than the normal crystalline array. The apparent termination of the R8 neurons at the edge of the medulla (M1) rather than at the normal M3 layer and the shape of the R7/R8 cells were very similar to their appearance at about 40 h APF before the second stage of R7/R8 targeting (Ting et al., 2005). Also, the medulla neuropil of the JH-treated animals was only about 70% the thickness of the normal neuropil. Thus, the JH-treated R7/R8 neurons did not complete their normal adult development.

### Normal onset of GAL4 expression in the OL interneurons

**Fig. 2.**
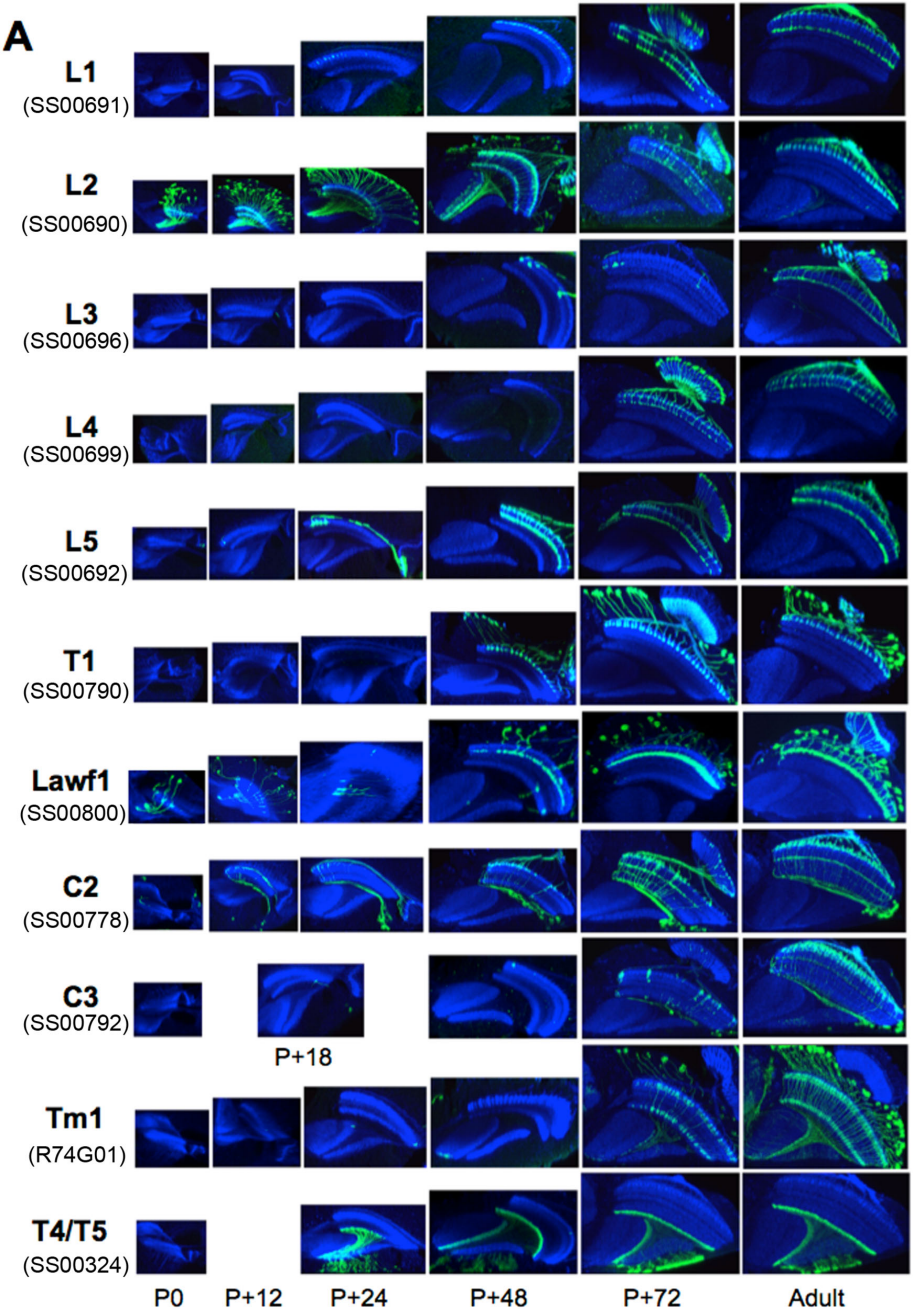

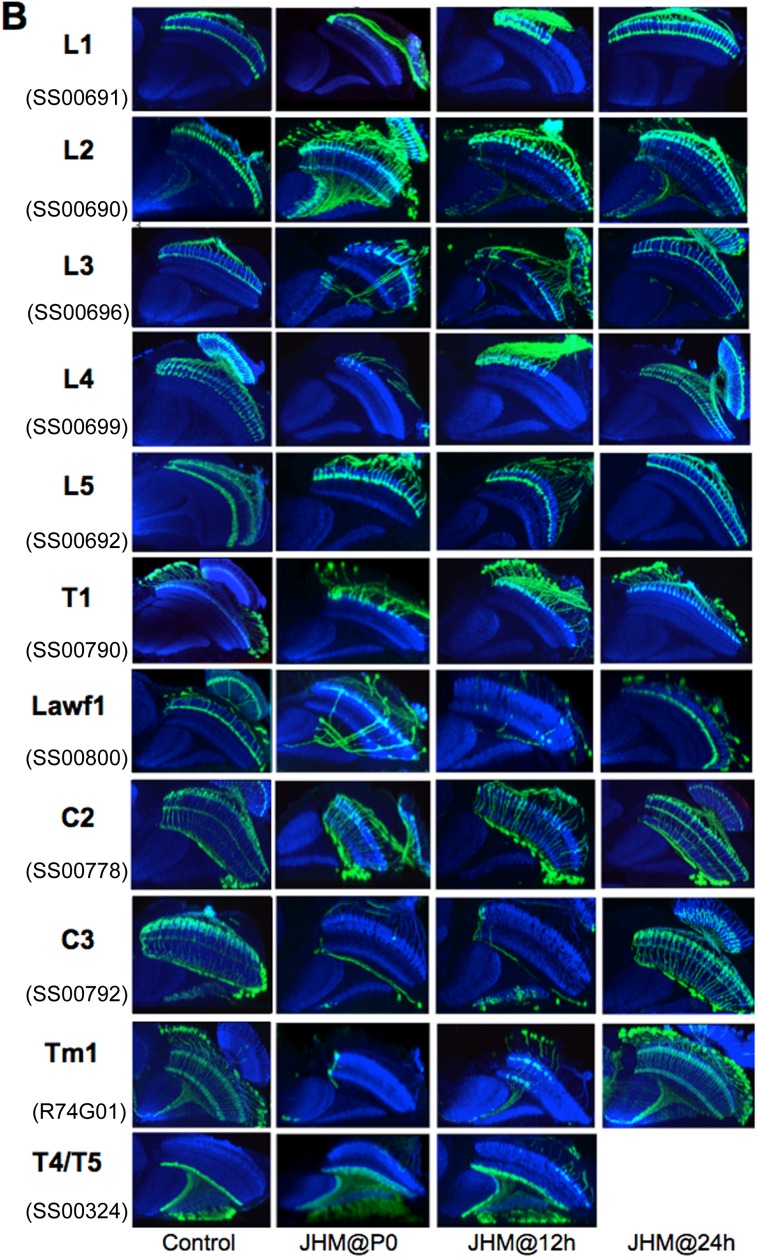
**The adult development of selected optic lobe neurons and the effect of JHM on that development.** (A) The normal adult development of different optic lobe neurons imaged using a myristylated GFP reporter. The stable split line number for each driver (see Table 1) is indicated. Times are hours after pupariation. Images are typical of three to five animals at each time point except for adults where 11-13 were imaged. (B) The effects of topical application of the JHM pyriproxifen at the onset (P0) and end (12 h APF) of the prepupal period and during the onset of adult development (24 h APF), on that development as assessed in the uneclosed day 1 (JHM at P0 and 12 h APF) or day 1 (JHM at 24 h APF) adults. Images are typical of six to nine animals treated at P0, four to seven at 12 h APF, and three to five at 24 h APF. The controls were untreated day 1 adults. The images are sections viewed along the dorsoventral axis. Blue, N-cadherin.

In our studies we used split-GAL4 intersection lines specific for different types of OL interneurons that arborize in the medulla. For most of the lines, there was no expression in the OL at the start of metamorphosis but the adult pattern began to appear at various times during adult differentiation (Fig. 2A). Some of the split lines showed early onset expression, such as seen for the L1, L5 and C2 neurons. In these cases, expression started by 12-24 h APF, but only in the oldest cells of each type. Progressively younger cells then began expressing as development progressed. Other lines showed a late onset of expression that was delayed until 48-72 h APF, then the cells began to express rather uniformly across the optic lobe, without regard to when they were born. All lines showed expression in day 0 adults (Fig. 2A).

### Effects of JHM treatment on GAL4 expression in OL interneurons

We topically applied JHM at various times and then assessed the effect in the pharate adult (for treatments at pupariation or 12 h APF since the treated animals do not eclose) or in newly emerged adults (for treatment at 24 h APF). On a gross level, treatment with JHM at the start of metamorphosis resulted in a thinning of the medulla (Fig. 1C), but the columnar organization was still evident.

Fig. 2B shows the response of OL interneurons to JHM treatment at pupariation or at 12-24 h thereafter and then examined at the end of adult development. We saw two major patterns of response. In one set of lines, such as those for C3, L1, L4, Tm1 and T1, treatment with JHM at P0 typically resulted in expression being seen in only a few neurons that were associated with columns at the anterior margin of the medulla. These would have been the oldest neurons in their class at the time of JH treatment. When treatment was delayed to 12 h APF, the boundary of expression moved to the medial region of the medulla whereas in animals treated at 24 h APF, all of the neurons expressed and the normal adult pattern was evident.

**Table 1.**
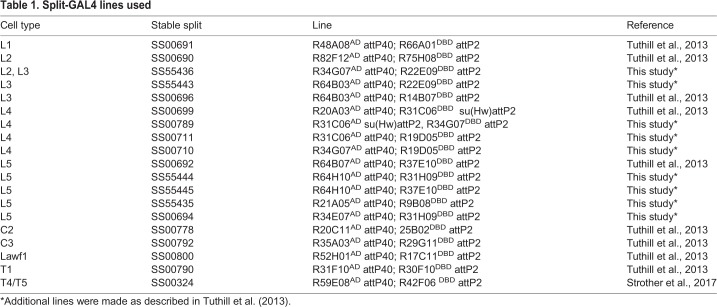
**Split-GAL4 lines used**

The second major pattern of response was evident in the driver lines for the L5, the C2, and the T4/T5 neurons. Unlike the above lines, these lines appeared to be insensitive to JH. Even after treatment with JHM at P0, the resultant adults showed a full pattern of expression across the length of the medulla. An atypical response was evident in the line for the L3 (SS00696) neurons. Early treatment with JH produced an inverted pattern with expression disrupted in the anterior medulla and uniform expression posteriorly. This was also evident after treatment at 12 h APF but full expression was evident after JHM at 24 h APF.

One question for JH-sensitive lines is whether the JHM treatment prevents the subsequent production of the neurons of that particular type, or, were the neurons produced but their GFP expression suppressed? We examined this question for the L1 and L4 neurons using Seven-up (Svp) [expressed in L1 neurons (Tan et al., 2015)], Brain-specific homeobox (Bsh; expressed in L4 and L5) and Dachshund (Dac; expressed in L1, L3, L4, L5) (Hasegawa et al., 2013) as markers. As shown in Fig. S1, these molecular markers showed that both the L1 and the L4 neurons are present in their normal positions across the lamina of the JHM-treated animals, although only the oldest of these neurons expressed GFP.

Two questions immediately arise from the above data: (1) can we identify features of a given driver line that determines whether or not it is JH sensitive, and (2) are cells inherently sensitive or insensitive, or is sensitivity a feature of just the drivers?

In terms of the first question, we see in Fig. 2B that the pattern of sensitivity of the various lines correlated strongly with their normal pattern of onset of GAL4 expression (Fig. 2A). Those lines that showed JH sensitivity in their expression typically showed a late expression that arose during the second half of adult development, appearing simultaneously across the medulla. By contrast, those lines that were JH-insensitive typically began expression during the first half of development and through this period their expression spread through the medulla columns correlated with their age. A notable exception to this generalization was the driver line used to mark the L1 neurons. This driver began expression in early adult development in a wave, but was sensitive to JHM.

**Fig. 3.**
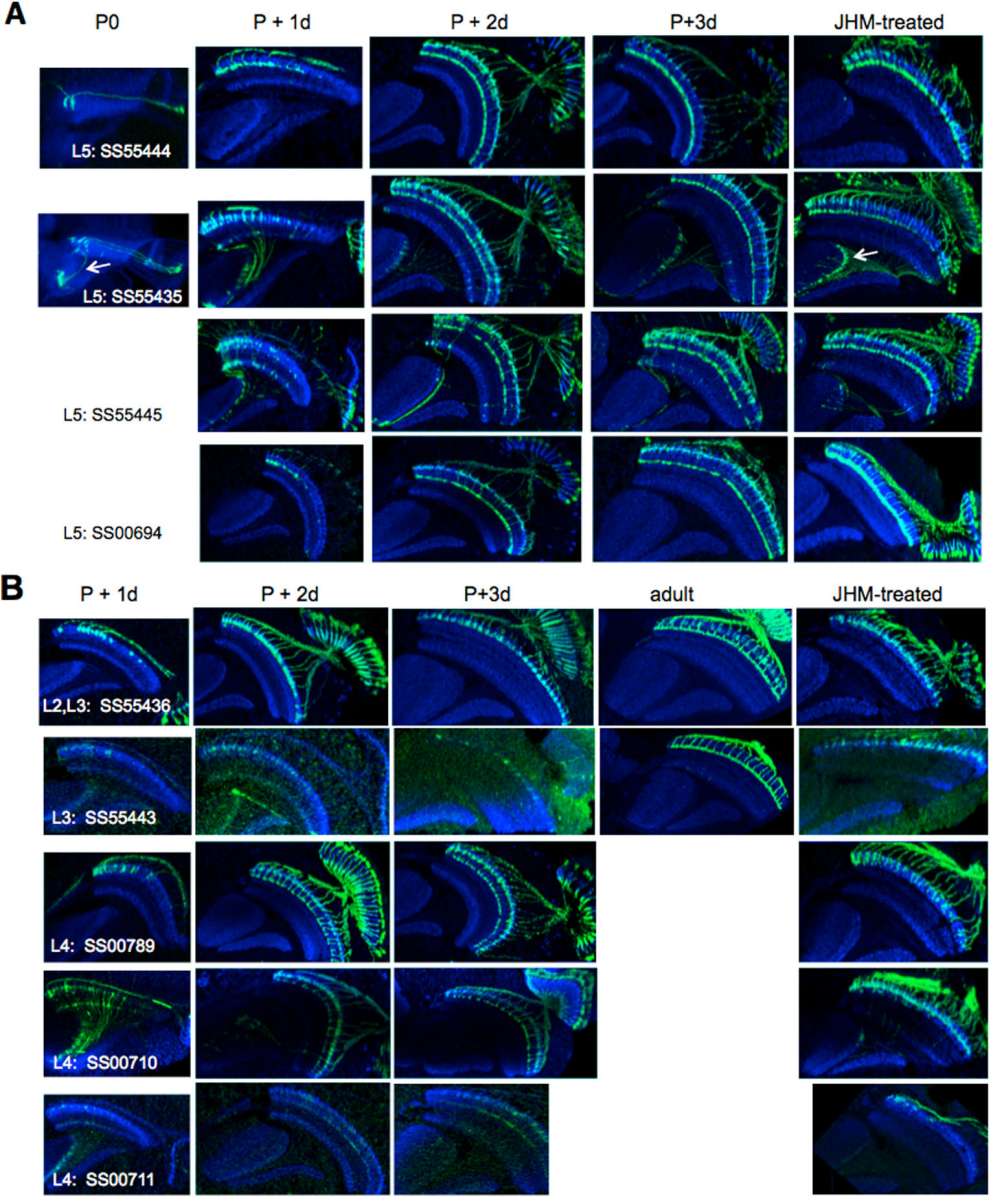
**The adult development of other drivers for the L5 (A) and the L2, L3 and L4 (B) neurons and the effect of JHM on that development.** The stable split line number for each driver is indicated. Treatment with the JHM pyriproxifen was about 8 h after pupariation and the effects assessed using a myristylated GFP reporter in uneclosed day 1 adults. Images for the developmental time series are typical of three animals per time point and for the JHM-treated animals of three to four per driver line. The images are sections viewed along the dorsoventral axis. The arrows in A point to the unidentified Tm neurons that expressed transiently during development but whose expression persisted in the adult after JHM treatment. Blue, N-cadherin.

The presence of the neurons in the JHM-treated animals suggested that at least part of the JH sensitivity rests at the level of the driver. Consequently, we chose a number of different driver lines for some of the neuron types to see if the response to JH was a driver or a cell phenomenon. We examined multiple driver lines with adult expression in most of the lamina monopolar neurons (Fig. 3). We typically examined the normal pattern of expression at one, two and three days after pupariation and the result of giving JHM at approximately 8 h APF. All four driver lines for the L5 neurons showed early onset expression (Fig. 3A). SS55444 and SS55435 were already expressing at P0 and had spread through half of the medulla columns by day 1. Lines SS55445 and SS00694 began expression after P0 and had spread through about a third of the medulla by day 1. All of these lines, though, were insensitive to JHM and showed expression across the entire medulla despite JHM treatment around 8 h APF.

Of the lines that expressed in the L3 neurons as adults (Fig. 3B, top), SS55436 was especially informative because the line also contained L2 neurons that showed early onset expression while the L3 neurons started expression between P+3 days and adult emergence, and both cell types were evident in the adult. However, in JHM-treated animals, only the L2 cells were evident. Flies of the SS55443 line were somewhat similar in that L2 neurons showed weak early expression, that then faded as development progressed to be replaced by L3 expression between P+3 days and the adult. In this case, JHM treatment similarly suppressed the late L3 expression and maintained the weak expression in L2 neurons.

Two of the lines that showed adult expression in the L4 neurons (Fig. 3B, bottom) – SS00789 and SS00710 – showed strong early expression already by day P+1 and expression was not suppressed by JHM treatment. By contrast, GFP expression in SS00711 came up after P+3 days and JHM treatment around P+8 h suppressed strong expression in the later-born L4 cells.

Therefore, regardless of the cell type we examined, the early expressing drivers were insensitive to treatment with JHM whereas the late expressing drivers were suppressed by JHM treatment.

### The effects of JHM treatment on the morphology of OL interneurons

The above section shows that drivers for a given neuron may differ in whether or not they are sensitive to treatment with JH. How does this sensitivity relate to the morphological response of the overall cell itself? In order to track the sensitivity of a neuron to JH, it is best to use a line with a driver that is JH-insensitive. This is best illustrated for the L5 lamina neurons. These cells project into the medulla with a terminal arbor in layer 5 and a second arbor in layer 1 and extending into layer 2 (Fischbach and Dittrich, 1989; Fig. 1A). The different arbors of L5 appear sequentially during pupal development (Fig. 2A; Nern et al., 2008). For animals treated at P0, the L5 neurons have produced their terminal arbors across the length of the medulla but showed a distal arbor in layer 1 in only a few of the oldest cells. For animals treated at 12 h APF, the distal arbor was evident in the oldest half of the L5 neurons and it is seen in all of the neurons in flies that had been treated with JHM at 24 h APF (Fig. 2B). Similar effects of JHM on the distal arbor were seen in the four other L5 driver lines assayed (Fig. 3A).

In the JHM-treated L4 driver line SS00789, the cells grew into the medulla, but both arbors showed morphological disruption (Fig. 3B). In contrast, in the L4 driver line SS00710 (Fig. 3B), the distal arbor in layer 2 was insensitive to JHM whereas the proximal arbor in layer 4 was sensitive so that after JHM treatment at about 8 h APF, only a few of the oldest cells had the proximal arbor. This difference in response between the two drivers could be due to the expression in line SS00710 of some L2 cells as well in L4 cells during development (as seen in the P+2 d image) and the L2 expression was maintained by the JHM treatment.

**Fig. 4.**
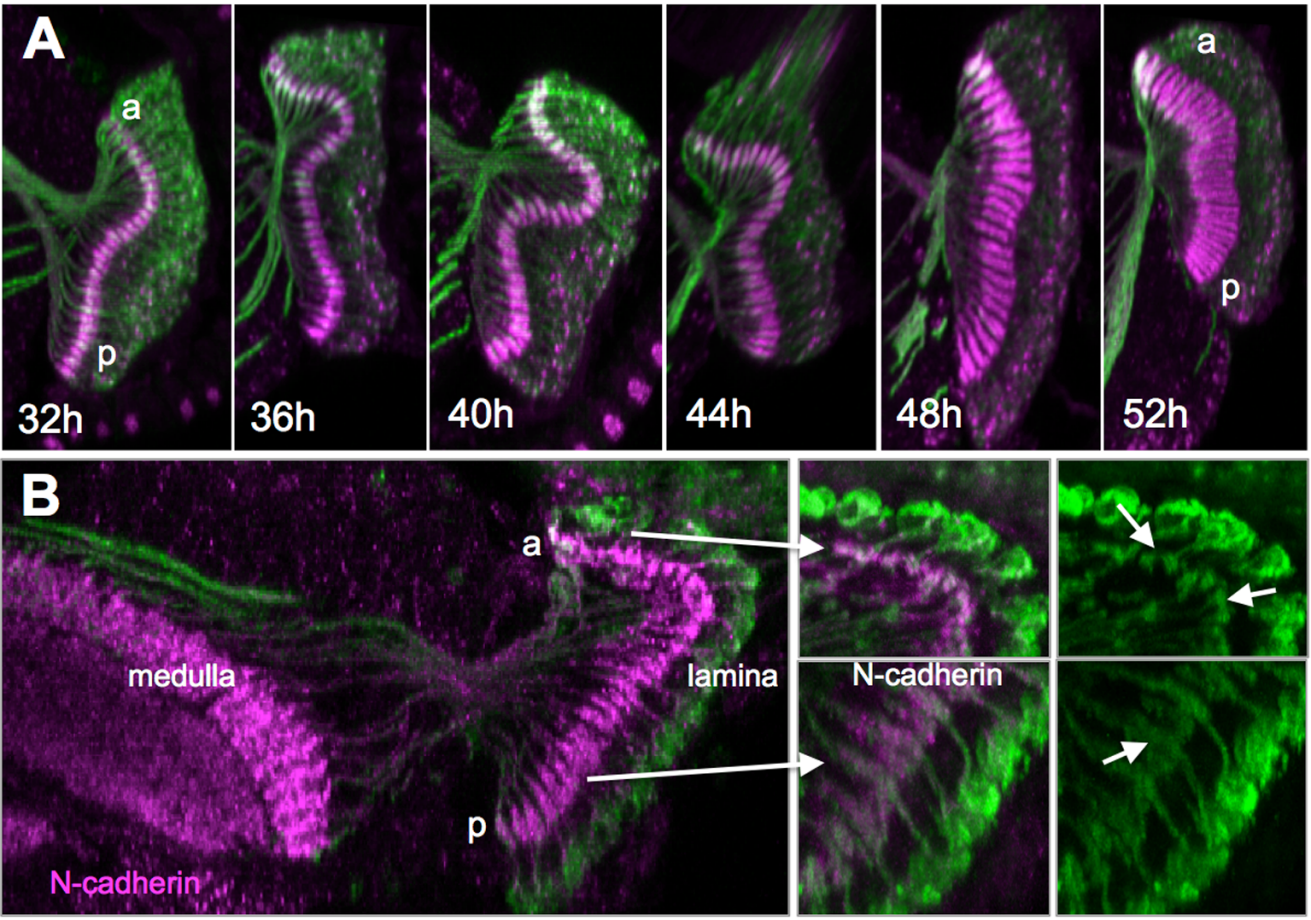
**Effect of JHM on the development of the dendritic neuropil of the L1-L3 lamina neurons, using the R27G05 pan-lamina line.** (A) Expansion of the lamina neuropil occurs simultaneously along its anterior (a)-posterior (p) axis. (B) The effect of JHM around 8 h APF on the expansion of the lamina neuropil. The inset shows neurons at the anterior (a) and posterior (p) ends. The small arrows show that the ones at the posterior have expanded whereas those at the anterior have not. Images are typical of three animals per time point.

Most of the synapses in the adult lamina are between the photoreceptors and lamina monopolar neurons L1-L3. The sprouting of the lamina cell dendrites results in a dramatic thickening of the lamina neuropil between 44-48 h APF (Fig. 4), and the photoreceptor axons subsequently make connections with these dendrites (Chen et al., 2014) . Interestingly, the expansion of this neuropil occurs simultaneously along the anterior-posterior axis of the neuropil in contrast to the posterior-anterior wave that characterized axon ingrowth and formation of terminal arbors. Treatment with JHM at P0 apparently suppressed dendritic elaboration as the lamina neuropil remained collapsed (Fig. 4).

### The effects of JHM treatment on the shutting off of driver expression during development

Three of the driver lines had neurons that showed an ‘early transient’ expression: they showed GFP expression at the start of metamorphosis but then lost expression by the end of adult development. As seen in Fig. 2A, this ‘early transient’ expression is seen in the SS00690 line that expressed strongly in immature unidentified Tm cells at the start of metamorphosis but then switched over to the L2 lamina neurons in the adult. Line SS00800 showed expression in scattered unidentified Tm neurons at the start of metamorphosis but only in the Lawf1 cells in the adult. Similarly, in the L5 SS55435 line, both L5 and unidentified Tm (likely Tm1/Tm2) cells were expressing at P0, but only L5 cells in the adult (Fig. 3A; see the adult image in Fig. 8). The expression in the Tm neurons in the L5 and Lawf1 lines was lost relatively early in adult development, whereas that in the Tm cells in the L2 line declined somewhat later after 48 h (Figs 2B and 3A). For all three lines, early treatment with JHM resulted in the persistence of expression in the Tm cells into the adult stage (Figs 2B and 3A).

### JH, Krüppel-homolog (Kr-h1) and adult development of the optic lobe

**Fig. 5.**
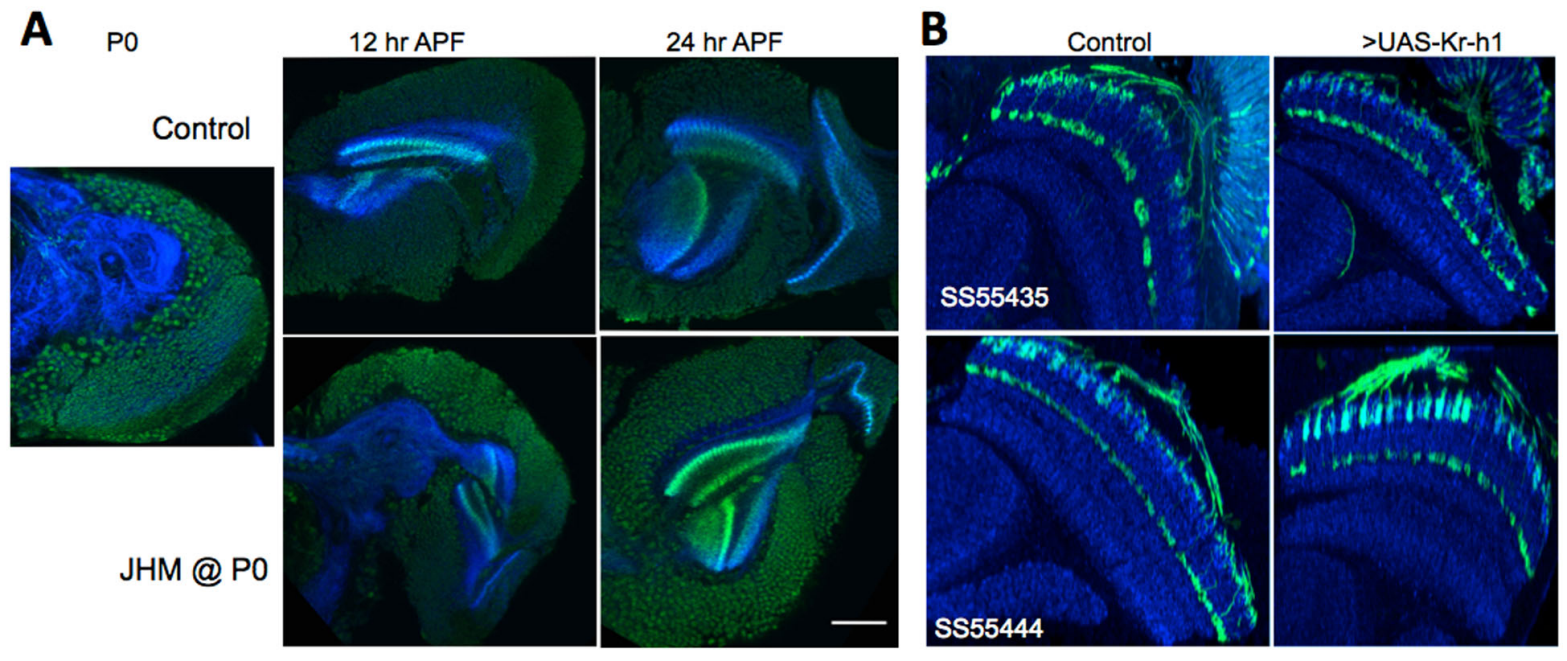
**The effects of JHM on Kr-h1 expression and the effects of Kr-h1 on lamina neuron development.** (A) Expression of Kr-h1 in the optic lobe at pupariation (P0) and 12 and 24 h thereafter as detected by immunocytochemical staining in control and animals treated with the JHM pyriproxifen at pupariation (P0). Images are typical of nine animals at P0 and 5 at P12 and P24 h. Scale bar: 75 µm. (B) The effect of misexpression of Kr-h1 in two L5 driver lines (SS55435, top, and SS55444, bottom) during metamorphosis from wandering to adult eclosion (*n*=4). Images of day 1 adult optic lobes are sections viewed along the dorsoventral axis of each line using a myristylated GFP reporter. Blue, N-cadherin.

In the immature stages of several insect orders, Kr-h1 has been found to be a direct target of the JH-*Met* complex and is necessary to prevent premature metamorphosis (Jindra et al., 2013, 2015). In *Drosophila*, Kr-h1 is present during larval life up to pupariation, but then rapidly disappears (Pecasse et al., 2000). Fig. 5A shows that Kr-h1 is present in both the brain and the optic lobe at pupariation but has disappeared from the OL nuclei by 12 h later. When JHM was given at pupariation, Kr-h1 expression persisted through at least 24 h APF (Fig. 5A). Therefore, Kr-h1 expression continues to serve as a molecular read-out of JH action in the developing OL.

We tried sustained, ectopic expression of Kr-h1 in OL neurons to see if maintenance of this transcription factor could mimic the effects of JH treatment. Initial attempts involving the pan-lamina GAL4 line R27G05 that is expressed at pupariation and thereafter resulted in embryonic lethality, so we selectively expressed Kr-h1 in L5 neurons using two lines that start expression by pupariation (SS55435 and SS55444). However, sustained expression of Kr-h1 in these cells did not result in a JH-like phenotype. The cells showed their normal production of distal and proximal arbors in the appropriate regions of the medulla (Fig. 5B). Therefore, the JH-induced persistence of Kr-h1 in the neurons is likely not the basis of the disruption of neuron development by JH.

### JH, the ecdysone receptor and Broad-Z3 during optic lobe development

**Fig. 6.**
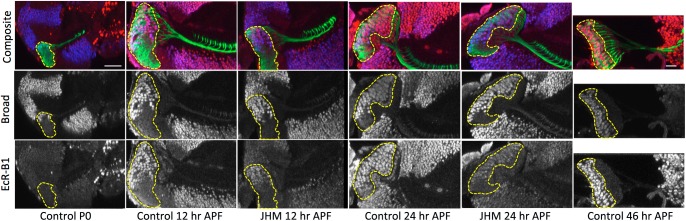
**The effect of JHM given at pupariation on EcR-B1 and Br-Z3 expression in the lamina during adult development.** The JHM pyriproxifen was applied at pupariation (P0) and images taken at 12 and 24 h thereafter of both the JHM-treated and the untreated controls. The control is also shown at 46 h APF. The top composite images show the pan-lamina line R27G05-GAL4 driving a myristylated GFP reporter in all the monopolar lamina neurons (L1-L5) and immunocytochemical staining for both EcR-B1 (red) and Br-Z3 (blue). The middle and bottom rows show the gray scale images of the Broad (Br-Z3) and EcR-B1 immunostaining respectively. Images are typical of three to four control animals and five JHM-treated animals for each time point. The images are sections viewed along the dorsoventral axis. The lamina neuron cell body region is indicated by the yellow dashed line. Scale bars: 50 µm (the one in the Control P0 column pertains to only that column; the one in the Control P+46 h column pertains to the remainder of the images).

Two hormone-related genes that are notable in their expression in the developing optic lobe are the B1 isoform of the ecdysone receptor (EcR-B1) and the *broad* transcription factor that underlies hormone-induced pupal commitment (Zhou and Riddiford, 2001). Our previous studies showed that EcR-B1 is up-regulated in the optic lobe beginning about 12 h APF and is maintained at high levels of expression until about 48 h APF (Truman et al., 1994; Riddiford et al., 2010). This expression is suppressed if JHM is given at the time of pupariation (Riddiford et al., 2010). The Z3 isoform of Broad (Br-Z3) is notable because it appears in the late larva in the oldest population of immature medulla neurons and gradually spreads into younger cells until the entire medulla is expressing by about 14 h APF where it remains until at least 27 h APF (Zhou et al., 2009). We examined the effects of JH on EcR-B1 and Broad-Z3 expression in lamina neurons using the pan-lamina neuron driver, R27G05 (Fig. 6). At P0 Broad-Z3 is expressed in only a few lamina neurons that are furthest from the proliferation zone and correspond to those cells that have already extended their axons into the medulla. This is consistent with Broad expression in other regions of the CNS in which expression begins after neurons have completed axon outgrowth and reached their initial targets (Zhou et al., 2009). By 12 h APF the Br-Z3 expression had spread to the oldest 50% of the lamina neurons, while at 24 h APF Br-Z3 was expressed by over 80% of the cells. Hence, Br-Z3 expression appears to occur in lamina neurons at a fixed time after their birth and likely corresponds to when they have finished axon outgrowth and reached their initial targets.

The expression of EcR-B1 differs from that of Br-Z3 in that it is absent at P0 (Fig. 6). EcR-B1 expression appears abruptly in OL neurons between 10-12 h APF (Truman et al., 1994) but in the lamina it is confined to the Br-Z3+ neurons. The same was seen at 24 h APF. Hence, the expression of EcR-B1 appears to be influenced by two factors: (1) the cells have acquired a particular level of maturation (signified by Br-Z3 expression) and (2) there is another factor that begins at about 10 h APF. As discussed below, the latter is the ecdysteroids that drive adult differentiation.

Treatment of animals with JH had no effect on the timing of Br-Z3 appearance in the lamina, but it markedly suppressed the levels of EcR-B1 expression, although the timing of its appearance was normal. In the context of optic lobe development, then, Br-Z3 expression appears to be linked to the wave of generation of OL neurons and appears at a specific early stage in the development of these cells, whereas that of EcR-B1 is responsive to hormonal signals such as ecdysone and JH.

**Fig. 7.**
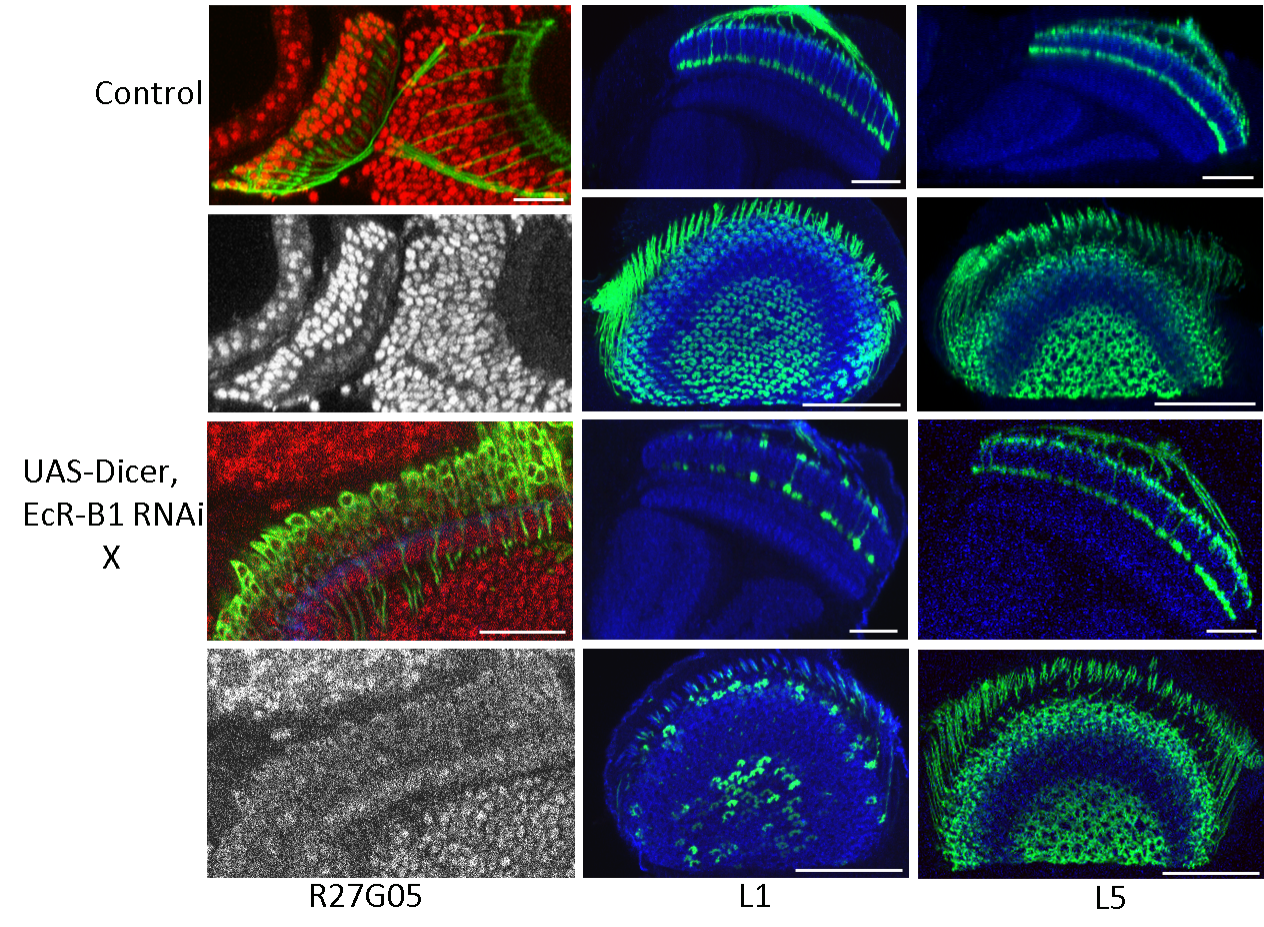
**The effect of the suppression of EcR-B1 on the development of lamina neurons L1 and L5.** The left-hand column shows the myristylated GFP expression of the pan-lamina lines R27G05-GAL4 (Control) and R27G05-GAL4>UAS-Dicer; EcR-B1 RNAi at 39 and 30 h respectively after pupariation together with the gray scale images of the EcR-B1 immunostaining in each. Images typical of three animals each. The next two columns are images of L1 (SS00691) and L5 (SS00692) neurons respectively in normal day 1 adults (top two rows) or in day 1 adults in which Dicer; EcR-B1 RNAi had been expressed at 29°C from the onset of wandering to eclosion (bottom two rows). Images typical of nine animals for L1 and 6 for L5. Images in the left-hand column and in the first and third rows are sections viewed along the dorsoventral axis and those in the second and fourth rows are viewed along the medial-lateral axis. Red, EcR-B1; blue, N-cadherin. Scale bar: 50 µm (25 µm for the R27G05, EcR-B1 RNAi at 30 h APF).

**Fig. 8.**
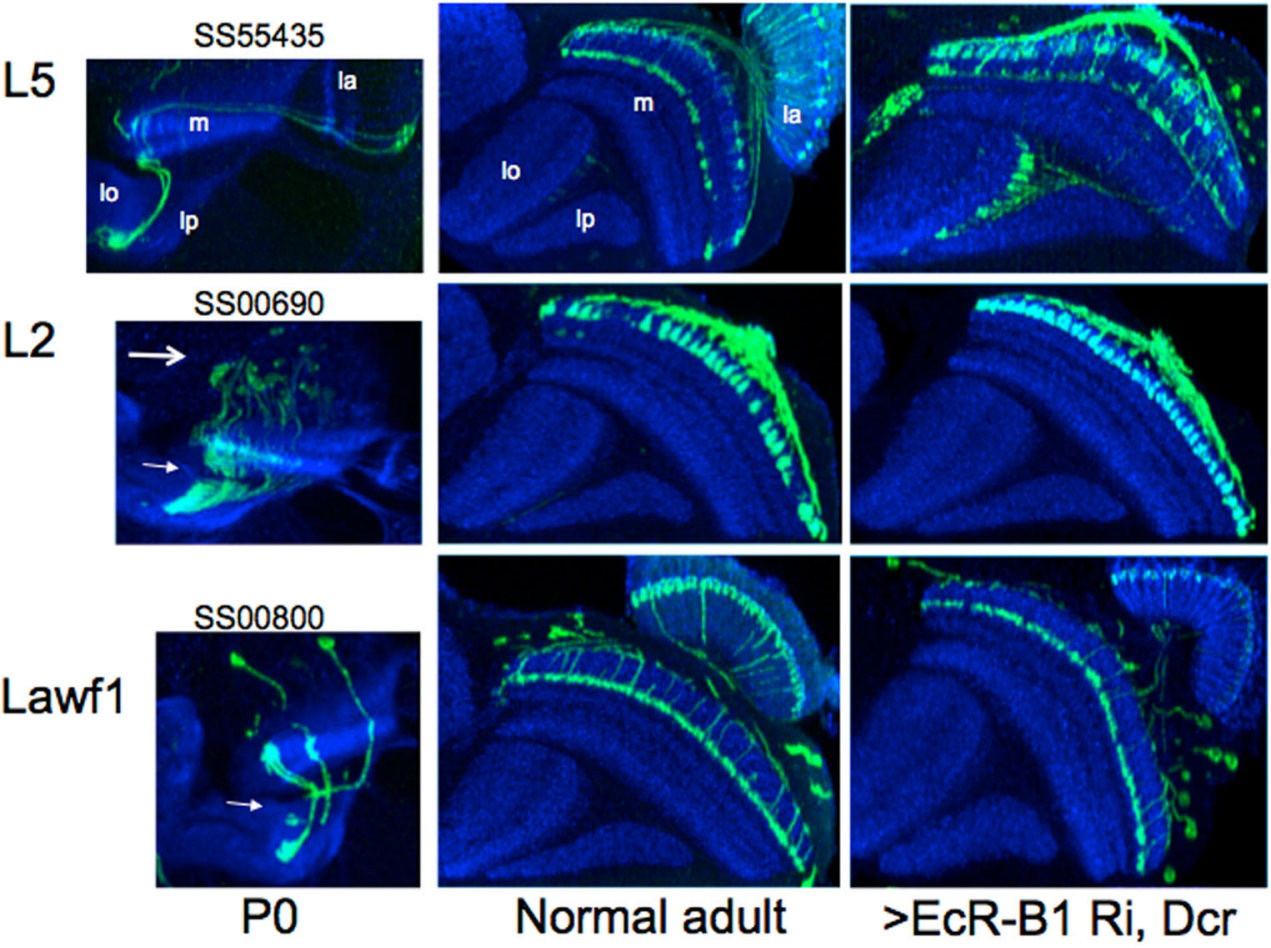
**The effect of EcR-B1 RNAi on the transient early expression of unidentified neurons.** Suppression of EcR-B1 prevents the turning-off of transient early expressing neurons in the L5 (SS55435) driver split-GAL4 line but not in the L2 (SS00690) lamina or Lawf1 (SS00800) lines. Dicer, EcR-B1 was expressed as described in Fig. 7. Images typical of six animals for L5, four for L2 and three for Lawf1. Images are sections viewed along the dorsoventral axis. la, lamina; lo, lobula; lp, lobula plate; m, medulla. The arrows in the P0 images indicate unidentified Tm neurons.

To determine whether the effect of exogenous JH at the time of pupariation on the development of the lamina neurons was due to suppression of EcR-B1 expression, we drove EcR-B1 RNAi and Dicer in three lines. When UAS-Dicer, EcR-B1 RNAi was expressed in the pan-lamina line R27G05, no EcR-B1 was subsequently seen in the lamina neurons at 30 h APF (Fig. 7, bottom left), a time when EcR-B1 is normally high (Fig. 7, top left; Fig. 6). When EcR-B1 was suppressed only in the L1 neurons from approximately 24 h APF onwards (see Fig. 2A for the timing of the onset of expression of the L1 driver), there was a marked reduction of the number of L1 neurons that expressed in the adult (Fig. 7). In contrast, the suppression of EcR-B1 in the L5 lamina neurons during the same time period had no effect on the levels of expression (Fig. 7). When EcR-B1 RNAi was expressed in the L5 neurons using the SS55435 L5 line in which the driver was already being expressed at P0 (Fig. 3A), the effect on the maturation of the L5 neurons was minimal (Fig. 8). This particular line also had the unidentified Tm neurons showing early transient expression, being evident at P0 but no longer expressing in the adult (Fig. 3A). Expression of EcR-B1 RNAi in this line prevented the shutting off of expression in these neurons so that they were still evident in the adult (Fig. 8). This maintenance of expression mimicked that seen when JH was given early in the prepupal period (Fig. 3A). To determine whether this effect of EcR-B1 RNAi was typical of drivers that showed early transient expression, we tested two other drivers (L2 and Lawf1) that also had this pattern. In both cases, EcR-B1 RNAi expression did not prevent their loss of expression during adult development (Fig. 8) whereas exposure to JHM at pupariation did (Fig. 2B). Overall, it appears that knockdown of EcR-B1 can mimic some of the actions of JH on OL development, but only to a limited extent.

## DISCUSSION

Unlike in most insects where JH is necessary to prevent metamorphosis and to maintain the larval state, in *Drosophila* the lack of JH during the final larval instar causes a reduced growth rate but does not interfere with normal development to pupariation (Mirth et al., 2014), but after pupariation the animals abruptly die around the time of head eversion (pupation) (Riddiford et al., 2010). Examination of the JH's role in visual system development showed that JH is critical for normal development of the optic lobe during the prepupal period. In its absence during the pupariation peak of ecdysteroid that initiates this development, the OL developed precociously in terms of the premature cessation of proliferation, appearance of EcR-B1, and extension of R7 beyond R8 in the medulla (Riddiford et al., 2010). Indeed, events that would normally start in the late prepupa as ecdysteroid titers are rising in the absence of JH, are now advanced to the earlier ecdysone peak, when JH is normally present. While these events in the OL would not cause lethality, we suggest that throughout the pupa there is precocious initiation of cell death programs that results in abrupt lethality.

The nervous system is an important tissue for studying JH action early in metamorphosis because, except for Malphigian tubule cells, neurons are the only larval cells that survive metamorphosis. The visual system is especially informative in this regard because of the constraints imposed by eye formation that involves the progressive production of rows of ommatidia as the morphogenetic furrow travels anteriorly across the eye imaginal disc. The resulting wave of photoreceptor ingrowth is correlated with corresponding waves of birth and differentiation of neurons across the first two neuropils of the OL, the lamina and the medulla.

Although JH must be present during the onset of the prepupal period to prevent the precocious recruitment of metamorphic programs, the present study shows that it must be absent during the latter part of the prepupal period and early adult development for subsequent events to occur normally. The application of JH at this time does not block metamorphic development, but results in nonviable adults that have a mixture of immature features (Restifo and Wilson, 1998). The wave of development that occurs across the visual neuropils provides a system in which, depending on the time of JH treatment, some cells are beyond their period of JH sensitivity, and hence are unaffected by treatment, whereas other cells of the same type have their development modified. We see these effects both at the level of overall cellular morphology and also in the expression state of various drivers.

### Exogenous JH effects on the developing visual system

Normally photoreceptors R7 and R8 grow into the medulla in an orderly progression from anterior to posterior as the morphogenetic furrow sweeps over the eye disc from posterior to anterior (Meinertzhagen and Hanson, 1993). R7 and R8 first grow into temporary layers in the medulla, but then between 40-60 h APF extend to their final medulla layers (Ting et al., 2005). Our data on the effects of JH given at pupariation show that whereas the initial photoreceptor ingrowth was undisturbed, the R-cell terminals did not complete the second stage of layer development and the tiling at their final destination was partially disrupted. The effects on tiling were similar to those on R7 tiling found by Ting et al. (2007) when activin signaling in R7 was disrupted. It has long been known since the studies of Ashburner (1970) and Postlethwait (1974), that the differentiation of the eye disc is unaffected by JH given at pupariation. Therefore, it is not surprising that the initial ingrowth of the JH-insensitive photoreceptors was unaffected. Their later extension deeper into the medulla is likely influenced by a JH-induced abnormal environment within the medulla.

Most of the interneurons that we examined showed some degree of JH sensitivity in their development. For the major photoreceptor targets in the lamina, L1-L3, their terminals in the medulla were well developed but they had severely suppressed dendrite elaboration in the lamina. The L5 neuron has no major arbor in the lamina but a distal arbor in layers 1 and 2 of the medulla and terminal arbors in layer 5. The terminal arbors were unaffected by JHM treatment whereas the distal arbor was blocked. Hence, these neurons show some features that are highly JH-sensitive and others that are JH-insensitive. The JH-insensitive features of the cell are the early features that occur in a wave correlated with the birth of the neurons. By contrast, the JH-sensitive features of the cells are later appearing features that, at least in the case of the L1-L3 dendrite formation, occur relatively uniformly across the A-P axis of the neuropil, regardless of the birth of the cell.

The relationship of JH sensitivity to early and late processes is also reflected in the driver lines. For the interneurons that innervated the medulla, there were basically two patterns in the response of drivers to JHM treatment. (1) Some drivers showed JH-sensitive expression (for example, those for L1, L4, C3, T1, and Tm1). After JHM treatment at P0, only the oldest neurons expressed the driver and progressively more neurons became competent to do so as the time of JHM treatment was delayed. One-third of the 21 lines studied had JH-sensitive drivers, and all but one of these lines (SS00691: L1) first expressed at 48 h APF or later and the expression of the driver appeared across the medulla synchronously. (2) Other drivers were insensitive to JHM treatment. All of these lines started expression early in metamorphosis and expression spread across the population in a wave that mimicked the birth of the cell population. A few drivers had uninterpretable patterns and L1 was notable in being the only driver that showed an early appearance but was also JH-sensitive. Besides the major group of drivers that we examined that expressed in the mature cells, we noted a few drivers that expressed in only the immature form of particular neurons. These ‘early transient’ drivers showed JH sensitivity and maintained expression into the adult after treatment with JHM.

### Molecular events occurring in early optic lobe development

The molecular events occurring in the optic lobe during its early development also can be divided into two classes: hormone-independent and hormone-dependent. Our previous studies showed that Broad-Z3 expression sweeps as a wave across the optic lobe through the period when the morphogenetic furrow is moving across the eye disc (Zhou et al., 2009). In the medulla, Broad-Z3 first appeared in the most medial columns of the medulla; but as the furrow progressed, it spread laterally as a wave so by 14 h APF just after the furrow had reached the anterior of the eye disc, the entire medulla showed expression. The present study shows that Broad-Z3 appears in the oldest lamina monopolar neurons as they reach the medulla slightly prior to pupariation and then spreads through this cell group over the next 24 h as their axons eventually invade all of the medulla columns. Like the movement of the morphogenetic furrow in the eye, the spread of expression of Broad-Z3 through the optic lobes occurs as a steady process that appears to ignore the dramatic swings in the ecdysteroid titer that coordinates the timing of other metamorphic events. Also, the appearance of Broad-Z3 in the lamina neurons was unaffected by the presence of exogenous JH. Therefore, the up-regulation of Broad-Z3 in the optic lobe is a consequence of a developmental program that is largely independent of the hormonal milieu.

By contrast, EcR-B1 normally does not appear in the optic lobe until the pupal stage (Truman et al., 1994). Then it appears only in those cells that have been committed to metamorphosis by expressing Br-Z3 as seen in Fig. 6 for the lamina monopolar neurons. Thus, after its abrupt appearance between 10-12 h APF, EcR-B1 appears in additional cells in a wave that coincides with Br-Z3 expression. EcR-B1 then persists in optic lobe neurons through the first half of adult development until about 48 h APF when the remnants of a gradient across the neuropils are lost and cells are behaving as a developmental unit. The onset of EcR-B1 expression coincides with the small peak of ecdysteroid at pupation (head eversion) and its offset with the decline of the ecdysteroid titer for adult development (Handler, 1982). JH given during the prepupal period suppresses the appearance of EcR-B1 in the optic lobe (Riddiford et al., 2010 and Fig. 6). We examined the possibility that the effects of JH treatment were mediated through its suppression of EcR-B1 expression. However, the suppression of EcR-B1 by the use of RNAi did not completely mimic any of the morphological effects of JHM treatment. In terms of particular drivers, though, the knock-down of EcR-B1 reduced the number of L1 neurons that expressed the SS00691 driver in the adult OL, but it did not completely prevent expression as seen after JHM treatment. Interestingly, EcR-B1 RNAi did mimic the action of JH during the prepupal period in preventing the turning-off of an early transient driver in the L5 SS55435 line although similar effects were not seen in two other cases of expression of early drivers prolonged to the adult by JHM.

Some of the effects of JHM treatment appear to be related to the suppression of EcR-B1 expression, but this clearly is not the whole story. The obvious candidate for the molecular switch involved in JH's disruption of normal adult development of the optic lobe neurons is the induced persistence of Kr-h1 (Fig. 5A), a larval specifier (Ureña et al., 2014, 2016). Yet misexpression of Kr-h1 in two different L5 drivers had no adverse effect on their development (Fig. 5B). The main molecular linkage between JH and the suppression of OL development remains unknown at present.

### Conclusions

**Fig. 9.**
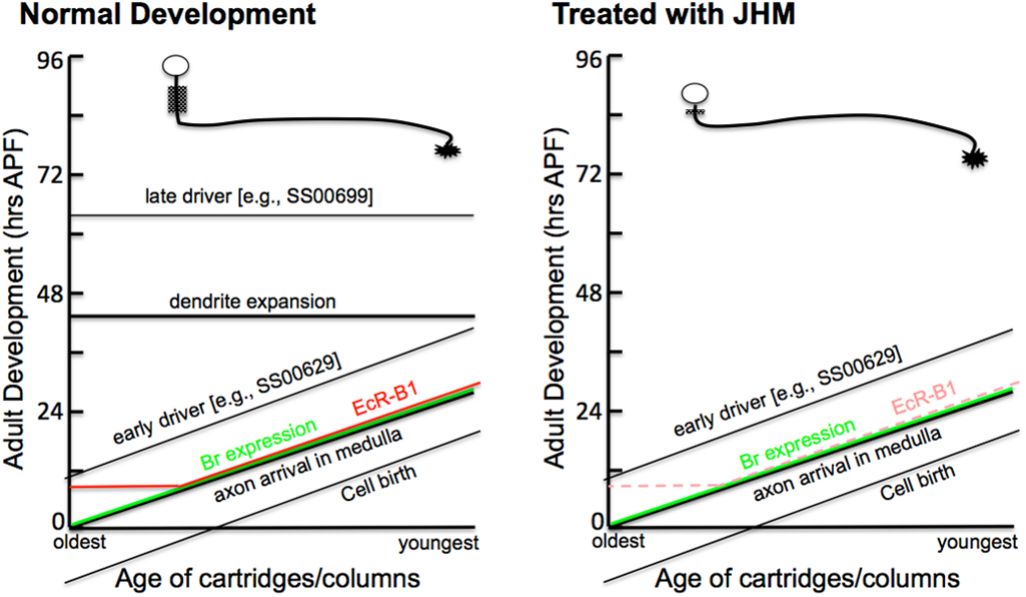
**Summary diagram of the effects of JHM at P0 on development of OL neurons.** See text for details. Br, Broad; EcR-B1, ecdysone receptor B1. Dashed line, suppressed expression.

The effect of ectopic JHM treatment on the development of the OL system is summarized in Fig. 9. There are two developmental programs at play early in metamorphosis: (1) a developmental wave of cell birth and differentiation that is linked to the development of the eye and (2) metamorphic events that are timed by titers of circulating ecdysteroids. There are morphological events (axon ingrowth), molecular expression (Br-Z3) and early driver expression that are part of this developmental wave and these are typically JH-insensitive. By contrast, other events such as dendritic expansion in the lamina monopolar cells and late driver expression are sensitive to JH and can be suppressed by ectopic JHM treatment early in metamorphosis. This JH sensitivity would be consistent with their presumed requirement for ecdysteroids for their occurrence. However, there is a substantial gap between the window of JH sensitivity and when these events occur. There obviously have to be JH-triggered events that span this gap. The L1 driver may be one pathway into this gap, being a relatively rare driver that comes on early and is also JH-sensitive. Also, EcR-B1 is likely part of this link, in that it appears in an ecdysone-dependent and JH-suppressible manner (starting at 10 h APF) but only in neurons that are old enough to be competent (reflected by Br-Z3 expression). However, EcR-B1 provides only part of this linkage.

## MATERIALS AND METHODS

### Fly stocks

Most of the neuron cell-type specific lines were split GAL4 lines made and previously used in the studies by Tuthill et al. (2013) and Strother et al. (2017) (Table 1). Additional split-GAL4 lines, which had not been used in previous studies, were constructed as described (Tuthill et al., 2013) (Table 1). For each split-GAL4 line, the activation domain (AD) and DNA-binding domain (DBD) hemidrivers and the ‘stable-split’ number (an identifier used by the Janelia split-GAL4 project) are indicated. The R74G01-GAL4 and R27G05-GAL4 lines (Jenett et al., 2012) were expressed in Tm1 neurons and in all lamina monopolar neurons, respectively. Other lines used were *UAS-EcR-B1* RNAi (Roignant et al., 2003), *P{EP}Kr-h1 Ep2289* (*UAS-Kr-h1*) (Abdelilah-Seyfried et al., 2000), *pJFRC12-10XUAS-IVS-myr::GFP attP2* (Pfeiffer et al., 2010), *UAS-nls-eGFP* (Stinger) (Barolo et al., 2000). The *UAS-Dicer*, *EcR-B1 RNAi*, *GFP* (*UAS-Dicer*; *pJFRC12-10XUAS-IVS-myr-GFPattP40*; *UAS-EcR-B1 RNAi/TM6B*); the *UAS-Kr-h1*, *GFP* (*P{EP}Kr-h1 EP2289*; *pJFRC12-10XUAS-IVS-myr::GFP attP2*); and the L1-GAL4>UAS-GFP (*pJFRC12-10UAS-IVS-myr GFP*; L1) stocks were made by Karen Hibbard in the Janelia Fly Core.

The fly stocks were all maintained on standard *Drosophila* medium at either 18°C or room temperature. The crosses to the UAS-myristylated GFP reporter line were maintained at 25°C under either constant light or 12L:12D. The crosses to the UAS-Dicer; EcR-B1 RNAi line and to the UAS-Kr-h1 line were placed at 29°C, 12L:12D at the onset of wandering.

### Juvenile hormone treatment

One hundred ng of the JH mimic (JHM) pyriproxifen (Sumitomo Chemical Co., Osaka, Japan) in 0.2 µl cyclohexane was applied to white puparia or at designated times thereafter.

### Immunocytochemistry and imaging

The brains and other tissues were dissected and fixed in 3.9% formaldehyde (Thermo Fisher Scientific) in phosphate-buffered saline (PBS; Mediatech, Manassas, USA) for 30 min to 1 h, then rinsed and incubated in PBS-1% Triton X (TX) with 2% normal donkey serum for 30 min. They were then incubated in PBS-1% TX with primary antibodies for 2-3 days at 4°C, repeatedly rinsed, then incubated with secondary antibodies overnight at 4°C. Primary antibodies used were: Broad-Z3 (mouse MAb Z3,9A7; 1:50), ecdysone receptor, B1 isoform (EcR-B1) (mouse MAb AD4.4; 1:50), chaoptin (mouse MAb 24B10; 1:50), N-cadherin (rat MAb DN-Ex#8; 1:50), svp (mouse MAb; 1:20), dac (mouse MAb dac2-3; 1:20), and elav (rat MAb 7E8A10 ; 1:100) (all from the Developmental Studies Hybridoma Bank, Iowa City, USA); Krüppel homolog (Kr-h) (Beck et al., 2005) (1:2000); Bsh (guinea pig polyclonal, 1: 200) (a gift from Larry Zipursky), and GFP (rabbit, 1:1000) (Life Technologies). Secondary antibodies were various Alexa Fluor 488-, 594-, or 647-conjugated donkey antisera raised against rabbit, mouse, or rat IgG fractions and used at 1:500 (Jackson Immunoresearch Laboratories, West Grove, USA). Immunostained preparations were typically dehydrated through a graded ethanol series, cleared in xylene and mounted in DPX (Sigma-Aldrich). Specimens shown in Fig. S1 were mounted in SlowFade Gold (Thermo Fisher Scientific, #S36937). The preparations were imaged on a Zeiss 510, 710 or 800 confocal microscope and processed with ImageJ (http://imagej.nih.gov/ij/).

## Supplementary Material

Supplementary information

## References

[BIO034025C1] Abdelilah-Seyfried S., Chan Y.-M., Zeng C., Justice N. J., Younger-Shepherd S., Sharp L. E., Barber S., Meadows S. A., Jan L. Y. and Jan Y. N. (2000). A gain-of-function screen for genes that affect the development of the *Drosophila* adult external sensory organ. *Genetics* 155, 733-752. 10835395 10.1093/genetics/155.2.733 PMC1461115

[BIO034025C2] Abdou M. A., Heb Q., Wen D., Zyaan O., Wang J., Xu J., Baumann A. A., Joseph J., Wilson T. G., Li S. et al. (2011). *Drosophila* Met and Gce are partially redundant in transducing juvenile hormone action. *Insect Biochem. Mol. Biol.* 41, 938-945. 10.1016/j.ibmb.2011.09.003 21968404

[BIO034025C3] Ashburner M. (1970). Effects of juvenile hormone on adult differentiation of *Drosophila melanogaster*. *Nature* 227, 187-189. 10.1038/227187a0 5428413

[BIO034025C4] Barolo S., Carver L. A. and Posakony J. W. (2000). GFP and beta-galactosidase transformation vectors for promoter/enhancer analysis in *Drosophila**.* *BioTechniques* 29, 726-732. 11056799 10.2144/00294bm10

[BIO034025C5] Baumann A. A., Texada M. J., Chen H. M., Etheredge J. N., Miller D. L., Picard S., Warner R., Truman J. W. and Riddiford L. M. (2017). Genetic tools to study juvenile hormone action in *Drosophila*. *Sci. Rep.* 7, 2132 10.1038/s41598-017-02264-4 28522854 PMC5437021

[BIO034025C6] Beck Y., Dauer C. and Richards G. (2005). Dynamic localisation of KR-H during an ecdysone response in *Drosophila*. *Gene Express. Patt.* 5, 403-409. 10.1016/j.modgep.2004.09.008 15661647

[BIO034025C7] Brennan K. A., Li T.-R., Bender M., Hsiung F. and Moses K. (2001). *Broad-complex*, but not *Ecdysone receptor*, is required for progression of the morphogenetic furrow in the *Drosophila* eye. *Development* 128, 1-11. 11092806 10.1242/dev.128.1.1

[BIO034025C8] Chen Y., Akin O., Nern A., Tsui C. Y. K., Pecot M. Y. and Zipursky S. L. (2014). Cell-type-specific labeling of synapses in vivo through synaptic tagging with recombination. *Neuron* 81, 280-293. 10.1016/j.neuron.2013.12.021 24462095 PMC4025979

[BIO034025C9] Fischbach K.-F. and Dittrich A. P. M. (1989). The optic lobe of *Drosophila melanogaster.* I. A Golgi analysis of wild-type structure. *Cell Tissue Res.* 258, 441-475. 10.1007/BF00218858

[BIO034025C10] Fristrom D. and Fristrom J. W. (1993). The metamorphic development of the adult epidermis. In *The Development of Drosophila melanogaster*, Vol. II (ed. Bate M. and Martinez-Arias A.), pp. 843-897. Plainview: Cold Spring Harbor Laboratory Press.

[BIO034025C11] Handler A. M. (1982). Ecdysteroid titers during pupal and adult development in *Drosophila melanogaster*. *Dev. Biol.* 93, 73-82. 10.1016/0012-1606(82)90240-8 6813165

[BIO034025C12] Hasegawa E., Kaido M., Takayama R. and Sato M. (2013). Brain-specific-homeobox is required for the specification of neuronal types in the *Drosophila* optic lobe. *Dev. Biol.* 377, 90-99. 10.1016/j.ydbio.2013.02.012 23454478

[BIO034025C13] Jenett A., Rubin G. M., Ngo T.-T. B., Shepherd D., Murphy C., Dionne H., Pfeiffer B. D., Cavallaro A., Hall D., Jeter J. et al. (2012). A GAL4-driver line resource for Drosophila neurobiology. *Cell Rep.* 2, 991-1001. 10.1016/j.celrep.2012.09.011 23063364 PMC3515021

[BIO034025C14] Jindra M., Palli S. R. and Riddiford L. M. (2013). The juvenile hormone signaling pathway in insect development. *Ann. Rev. Entomol.* 58, 181-204. 10.1146/annurev-ento-120811-153700 22994547

[BIO034025C15] Jindra M., Belles X. and Shinoda T. (2015). Molecular basis of juvenile hormone signaling. *Curr. Opin. Insect Sci.* 11, 39-46. 10.1016/j.cois.2015.08.004 28285758

[BIO034025C16] Kulkarni A., Ertekin D., Lee C.-H. and Hummel T. (2016). Birth order dependent growth cone segregation determines synaptic layer identity in the *Drosophila* visual system. *Elife* 5, e13715 10.7554/eLife.13715 26987017 PMC4846375

[BIO034025C17] Liu Y., Sheng Z., Liu H., Wen D., He Q., Wang S., Shao W., Jiang R.-J., An S., Sun Y. et al. (2009). Juvenile hormone counteracts the bHLH-PAS transcription factors MET and GCE to prevent caspase-dependent programmed cell death in *Drosophila*. *Development* 136, 2015-2025. 10.1242/dev.033712 19465595

[BIO034025C18] Meinertzhagen I. A. and Hanson T. E. (2003). The development of the optic lobe. In *The Development of Drosophila melanogaster*, Vol. II (ed. Bate M. and Martinez-Arias A.), pp. 1363-1491. Plainview: Cold Spring Harbor Laboratory Press.

[BIO034025C19] Mirth C. K., Tang H. Y., Makhon-Moore S. C., Salhadar S., Gokhale R. H., Warner R. D., Koyama T., Riddiford L. M. and Shingleton A. W. (2014). Juvenile hormone regulates body size and perturbs insulin signaling in *Drosophila*. *Proc. Nat. Acad. Sci. USA* 111, 7018-7023. 10.1073/pnas.1313058111 24778227 PMC4024895

[BIO034025C20] Nern A., Zhu Y. and Zipursky S. L. (2008). Local N-cadherin interactions mediate distinct steps in the targeting of lamina neurons. *Neuron* 58, 34-41. 10.1016/j.neuron.2008.03.022 18400161 PMC2692379

[BIO034025C21] Nijhout H. F. (1995). *Insect Hormones*. Princeton: Princeton University Press.

[BIO034025C22] Pecasse F., Beck Y., Ruiz C. and Richards G. (2000). *Krüppel-homolog*, a stage-specific modulator of the prepupal ecdysone response, is essential for *Drosophila* metamorphosis. *Dev. Biol.* 221, 53-67. 10.1006/dbio.2000.9687 10772791

[BIO034025C111] Pfeiffer B. D., Ngo T-T. B., Hibbard K. L., Murphy C., Jenett A., Truman J. W. and Rubin G. M. (2010). Refinement of tools for targeted gene expression in Drosophila. *Genetics* 186, 735-755. 10.1534/genetics.110.119917 20697123 PMC2942869

[BIO034025C24] Postlethwait J. H. (1974). Juvenile hormone and the adult development of *Drosophila*. *Biol. Bull.* 147, 119-135. 10.2307/1540573 4210845

[BIO034025C25] Restifo L. L. and Wilson T. G. (1998). A juvenile hormone agonist reveals distinct developmental pathways mediated by ecdysone-inducible Broad Complex transcription factors. *Dev. Genet.* 22, 141-159. 10.1002/(SICI)1520-6408(1998)22:2<141::AID-DVG4>3.0.CO;2-6 9581286

[BIO034025C26] Riddiford L. M. (1994). Cellular and molecular actions of juvenile hormone. I. General considerations and premetamorphic actions. *Adv. Insect Physiol.* 24, 213-227. 10.1016/S0065-2806(08)60084-3

[BIO034025C27] Riddiford L. M. (2012). How does juvenile hormone control insect metamorphosis and reproduction? *Gen. Comp. Endocrinol.* 179, 477-484. 10.1016/j.ygcen.2012.06.001 22728566

[BIO034025C28] Riddiford L. M., Truman J. W., Mirth C. K. and Shen Y. (2010). A role for juvenile hormone in the prepupal development of *Drosophila melanogaster*. *Development* 137, 1117-1126. 10.1242/dev.037218 20181742 PMC2835327

[BIO034025C29] Roignant J.-Y., Carré C., Mugat B., Szymczak D., Lepesant J.-A. and Antoniewski C. (2003). Absence of transitive and systemic pathways allows cell-specific and isoform-specific RNAi in *Drosophila*. *RNA* 9, 299-308. 10.1261/rna.2154103 12592004 PMC1370397

[BIO034025C30] Strother J. A., Wu S.-T., Wong A. M., Nern A., Rogers E. M., Le J. Q., Rubin G. M. and Reiser M. B. (2017). The emergence of directional selectivity in the visual motion pathway of *Drosophila*. *Neuron* 94, 168-182. 10.1016/j.neuron.2017.03.010 28384470

[BIO034025C31] Tan L., Zhang K. X., Pecot M. Y., Nagarkar-Jaiswal S., Lee P.-T., Takemura S., McEwen J. M., Nern A., Xu S., Tadros W. et al. (2015). Ig superfamily ligand and receptor pairs expressed in synaptic partners in *Drosophila*. *Cell* 163, 1756-1769. 10.1016/j.cell.2015.11.021 26687360 PMC4804707

[BIO034025C32] Ting C.-Y. and Lee C.-H. (2007). Visual circuit development in *Drosophila*. *Curr. Opin. Neurobiol.* 17, 65-72. 10.1016/j.conb.2006.12.004 17204415

[BIO034025C33] Ting C.-Y., Yonekura S., Chung P., Hsu S. N., Robertson H. M., Chiba A. and Lee C.-H. (2005). *Drosophila* N-cadherin functions in the first stage of the two-stage layer-selection process of R7 photoreceptor afferents. *Development* 132, 953-963. 10.1242/dev.01661 15673571

[BIO034025C34] Ting C.-Y., Herman T., Yonekura S., Gao S., Wang J., Serpe M., O'Connor M. B., Zipursky S. L. and Lee C.-H. (2007). Tiling of R7 axons in the *Drosophila* visual system is mediated both by transduction of an activin signal to the nucleus and by mutual repulsion. *Neuron* 56, 793-806. 10.1016/j.neuron.2007.09.033 18054857 PMC2693211

[BIO034025C35] Truman J. W., Talbot W. S., Fahrbach S. E. and Hogness D. S. (1994). Ecdysone receptor expression in the CNS correlates with stage-specific responses to ecdysteroids during *Drosophila* and *Manduca* development. *Development* 120, 219-234. 8119129 10.1242/dev.120.1.219

[BIO034025C36] Truman J. W., Hiruma K., Allee J. P., MacWhinnie S. G. B., Champlin D. T. and Riddiford L. M. (2006). Juvenile hormone is required to couple imaginal disc formation with nutrition in insects. *Science* 312, 1385-1388. 10.1126/science.1123652 16741122

[BIO034025C37] Tuthill J. C., Nern A., Holtz S. L., Rubin G. M. and Reiser M. B. (2013). Contributions of the 12 neuron classes in the fly lamina to motion vision. *Neuron* 79, 128-140. 10.1016/j.neuron.2013.05.024 23849200 PMC3806040

[BIO034025C38] Ureña E., Manjón C., Franch-Marro X. and Martin D. (2014). Transcription factor E93 specifies adult metamorphosis in hemimetabolous and holometabolous insects. *Proc. Natl. Acad. Sci. USA* 111, 7024-7029. 10.1073/pnas.1401478111 24778249 PMC4024875

[BIO034025C39] Ureña E., Chafinao S., Manjón C., Franch-Marro X. and Martin D. (2016). The occurrence of the holometabolous pupal stage requires the interaction between E93, Krüppel-homolog 1 and Broad-complex. *PLoS Genet.* 12, e1006020 10.1371/journal.pgen.1006020 27135810 PMC4852927

[BIO034025C40] Wolff T. and Ready D. F. (1993). Pattern formation in the *Drosophila* retina. In *The Development of Drosophila melanogaster*, Vol. II (ed. Bate M. and Martinez-Arias A.), pp. 1277-1325. Plainview: Cold Spring Harbor Laboratory Press.

[BIO034025C41] Zhou B. and Riddiford L. M. (2001). Hormonal regulation and patterning of the Broad-Complex in the epidermis and wing discs of the tobacco hornworm, *Manduca sexta*. *Dev. Biol.* 231, 125-137. 10.1006/dbio.2000.0143 11180957

[BIO034025C42] Zhou X. and Riddiford L. M. (2002). Broad-Complex specifies pupal development and mediates the prevention of the pupal-adult transformation by juvenile hormone in *Drosophila* and *Manduca*. *Development* 129, 2259-2269. 11959833 10.1242/dev.129.9.2259

[BIO034025C43] Zhou B., Williams D. W., Altman J., Riddiford L. M. and Truman J. W. (2009). Temporal patterns of *broad* isoform expression during the development of neuronal lineages in *Drosophila*. *Neural Dev.* 4, 39 10.1186/1749-8104-4-39 19883497 PMC2780399

