# Supplementary material for: Juvenile hormone reveals mosaic developmental programs in the metamorphosing optic lobe of Drosophila melanogaster
Source: Biol Open. 2018 Apr 15;7(4):bio034025. doi: 10.1242/bio.034025 (PMC5936066; doi:10.1242/bio.034025)
Supplement: Supplementary information [file biolopen-7-034025-s1.pdf]

Fig. S1A

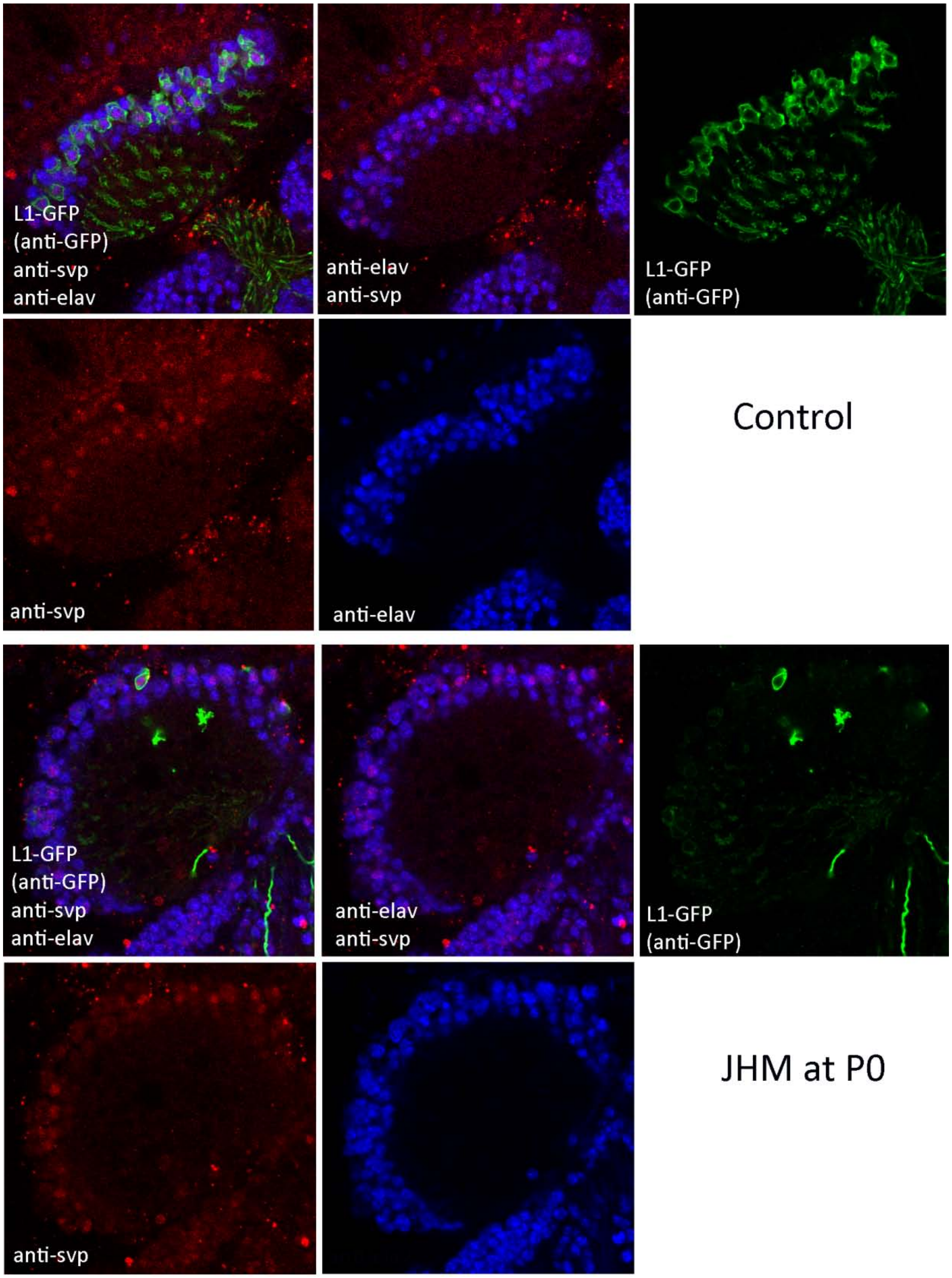

Biology Open • Supplementary information

Fig. S1B

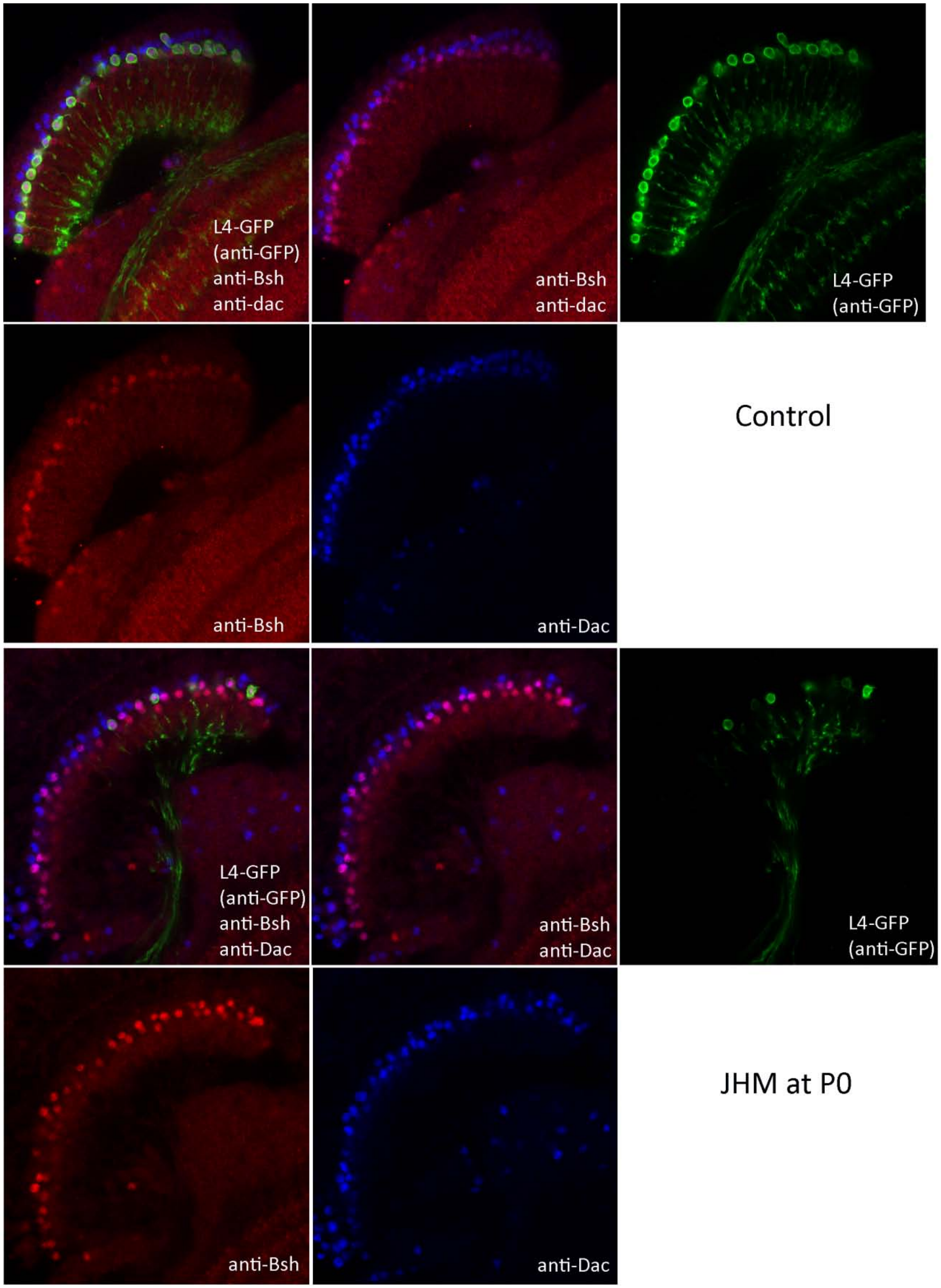

Biology Open • Supplementary information

Figure S1. The presence of lamina neurons in the adult after JHM treatment at pupariation. A) The presence of the L1 neurons in the adult lamina as indicated by their specific Svp staining and their Elav staining after treatment with the JHM pyriproxifen at P0. The L1 driver is expressed in only one of these cells as compared to the untreated Control.
B) The presence of L4 neurons in the adult lamina as indicated by their Bsh and Dac staining after treatment with the JHM pyriproxifen at P0. The L4 driver is expressed in only a few of these cells as compared to the untreated control. Bsh and Dac (also expressed in L1 and L3) are present in both L4 and L5 neurons but Dac expression appears weaker in L5 compared to L4 in both JH-treated and control cells. The images are sections viewed along the dorsoventral axis.
